# Virtual reality visual feedback for hand-controlled scanning probe microscopy manipulation of single molecules

**DOI:** 10.3762/bjnano.6.220

**Published:** 2015-11-16

**Authors:** Philipp Leinen, Matthew F B Green, Taner Esat, Christian Wagner, F Stefan Tautz, Ruslan Temirov

**Affiliations:** 1 Peter Grünberg Institut (PGI-3), Forschungszentrum Jülich, 52425 Jülich, Germany; 2 Jülich Aachen Research Alliance (JARA)-Fundamentals of Future Information Technology, 52425 Jülich, Germany

**Keywords:** non-contact atomic force microscopy (NC-AFM), Oculus Rift, perylene-3,4,9,10-tetracarboxylic dianhydride (PTCDA), scanning probe microscopy (SPM), scanning tunnelling microscopy (STM), single-molecule manipulation, virtual reality interface

## Abstract

Controlled manipulation of single molecules is an important step towards the fabrication of single molecule devices and nanoscale molecular machines. Currently, scanning probe microscopy (SPM) is the only technique that facilitates direct imaging and manipulations of nanometer-sized molecular compounds on surfaces. The technique of hand-controlled manipulation (HCM) introduced recently in *Beilstein J. Nanotechnol.* **2014,** *5,* 1926–1932 simplifies the identification of successful manipulation protocols in situations when the interaction pattern of the manipulated molecule with its environment is not fully known. Here we present a further technical development that substantially improves the effectiveness of HCM. By adding Oculus Rift virtual reality goggles to our HCM set-up we provide the experimentalist with 3D visual feedback that displays the currently executed trajectory and the position of the SPM tip during manipulation in real time, while simultaneously plotting the experimentally measured frequency shift (Δ*f*) of the non-contact atomic force microscope (NC-AFM) tuning fork sensor as well as the magnitude of the electric current (*I*) flowing between the tip and the surface. The advantages of the set-up are demonstrated by applying it to the model problem of the extraction of an individual PTCDA molecule from its hydrogen-bonded monolayer grown on Ag(111) surface.

## Introduction

The recently introduced scanning probe microscopy (SPM) technique of hand controlled manipulation (HCM) allows the operator of the SPM to manipulate single molecules on surfaces by coupling the motion of the microscope tip to the movements of the hand of the operator in 3D space [1]. The possibility to control the position of the SPM tip by hand is especially advantageous when the manipulation is performed in an environment where the forces acting on the manipulated molecule are a priori not fully known. In such cases, a hand-controlled trial and error search for successful manipulation protocols is more efficient than model-based simulation approaches, simply because in HCM tests of various manipulation protocols can be performed very quickly by execution and comparison of many alternative tip trajectories.

**Figure 1:**
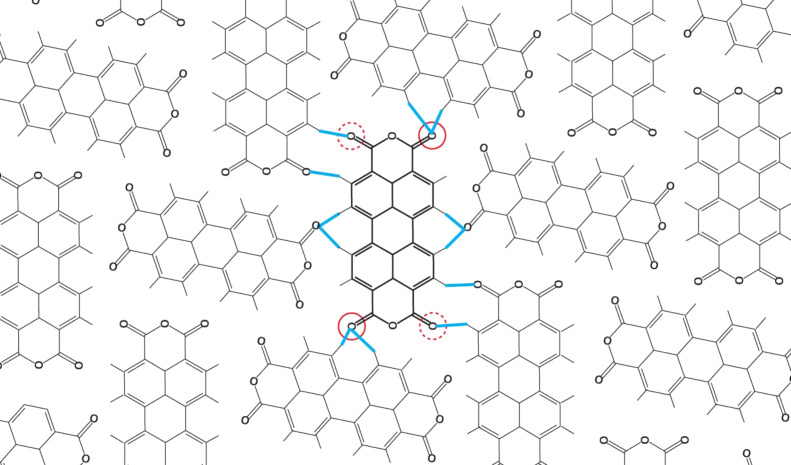
Structural model of a fragment of the commensurate PTCDA monolayer grown on an Ag(111). Blue lines mark the expected positions of intermolecular hydrogen bonds. Red circles mark positions of carboxylic oxygen atoms that may be used for contacting the molecule by the tip. Here we only contact the oxygen atoms marked by solid circles.

For its initial demonstration HCM was applied to the problem of extraction of single PTCDA molecules out of their commensurate monolayer grown on the Ag(111) surface [1–3]. Similar to the current study those experiments were performed with a commercial, combined qPlus tuning fork [4] non-contact atomic force/scanning tunnelling microscope (NC-AFM/STM) operated at 5 K under ultra-high vacuum conditions. Each extraction attempt started with positioning the tip over one of the four carboxylic oxygen atoms (marked by red circles in Figure 1) of the PTCDA molecule. The tip was then approached to the surface until the operator observed a sudden jump in the *I* and Δ*f* signals. Here the oxygen atom under the tip flipped up toward the tip apex, thereby establishing a chemical bond between the tip and the molecule [5–6]. Through this bond the molecule can be lifted from the surface by pulling it with the tip as it is retracted away from the surface. When the contacted molecule rests isolated on the surface, retracting the tip on a straight line away from the surface allows for the successful lifting of the molecule [7–8]. However if the contacted molecule resides inside an ordered PTCDA monolayer, most of the attempts of pulling it straight up fail [1], because intermolecular interactions in the monolayer hold the molecules together [9]. When the force exerted by the intermolecular bonds overcomes the strength of the tip–molecule bond, the latter breaks and the molecule being lifted drops back to the surface.

Previously, we employed HCM to identify tip retraction trajectories which minimize the instances of tip–molecule bond breaking that prematurely end the manipulation procedure [1]. The task was accomplished by extracting 48 PTCDA molecules one by one. The experiment started without any prior training of the operator and lasted for about 50 working hours. In the course of the experiment an average time between successful manipulation attempts decreased from 40 to 13 minutes, which suggested that the operator learned how to move the tip in order to extract the molecule successfully. The learning was achieved by observing and intuitively interpreting the real-time *I*(*t*) and Δ*f*(*t*) signals displayed during the manipulation on the screen of the oscilloscope, where a premature break of the tip–molecule contact was revealed as a simultaneous sharp drop of both *I*(*t*) and Δ*f*(*t*) signal values.

## Experimental

**Figure 2:**
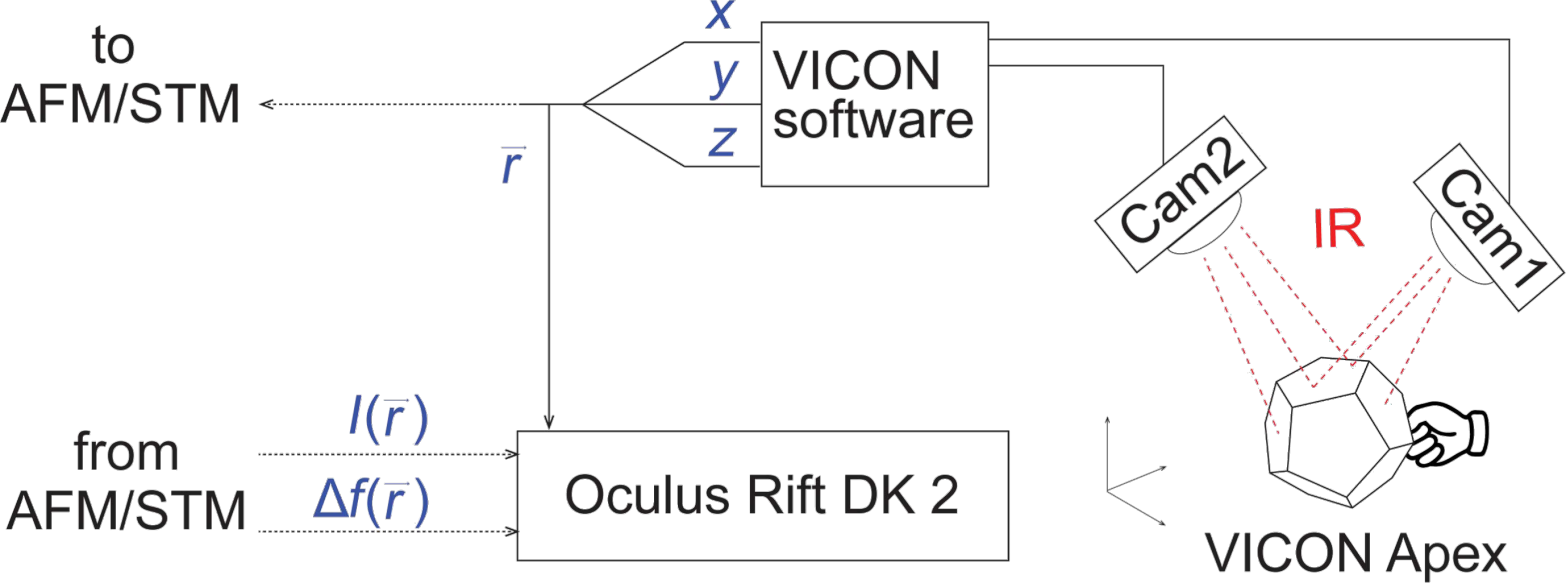
Scheme of the set-up used for HCM with a visual feedback using Oculus Rift virtual reality goggles (ORt). VICON 3D tracking system with two IR cameras follows the position of the VICON Apex device. VICON Apex has a dodecahedral shape, on the surface of which multiple sources of IR light are installed. The emitted IR light is registered by the cameras that compose an image from which the VICON software reconstructs the position and the orientation (not used in the experiment) of the Apex device in 3D space. The obtained coordinates *x*,*y*,*z* are used to set the position of the AFM/STM tip. The same coordinates are used to plot 3D trajectories in the virtual space of (ORt).

While in its present form HCM is able to generate many tip trajectories in a fast and intuitive manner, it does not yet allow for a convenient visual inspection of the generated data in real time. Having such a possibility would make the analysis of manipulation trajectory data much simpler. It would also help to transfer knowledge between different users and/or experiments, thus facilitating systematic learning during which manipulation protocols are refined and corrected in multiple steps. Visualization of the manipulation trajectory data should therefore greatly increase the effectiveness of HCM and extend the range of its possible applications. Here we introduce a system that visualizes HCM data in real time by displaying the actual tip position as well as the history of its movements in 3D using Oculus Rift DK 2 (ORt) virtual reality goggles. A scheme of the set-up is shown in Figure 2. As before we use two VICON Bonita cameras for 3D tracking of the position of the operator’s hand. In contrast to our previous work [1] where we tracked a single reflective marker we now use a VICON Apex device. Due to its shape and multiple active sources of infra-red (IR) light on its surface, tracking of the Apex device is more reliable [10]. Moreover, it allows for the implementation of additional service functions that help the operator to perform manipulation experiments without the use of a PC keyboard.

3D trajectories of the tip are visualised in ORt by intercepting the coordinates of the Apex device 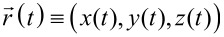 that are sent to the SPM electronics to position the tip [1] and forwarding them to ORt where a dot is plotted at each 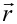, thus generating a 3D image of the tip trajectory. In addition, each dot is coloured to exhibit values of 
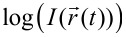
 or 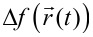 as obtained from the SPM. Switching between the log(*I*) and Δ*f* color-codes can be performed by pressing a custom-defined action button on the Apex device.

## Results and Discussion

**Figure 3:**
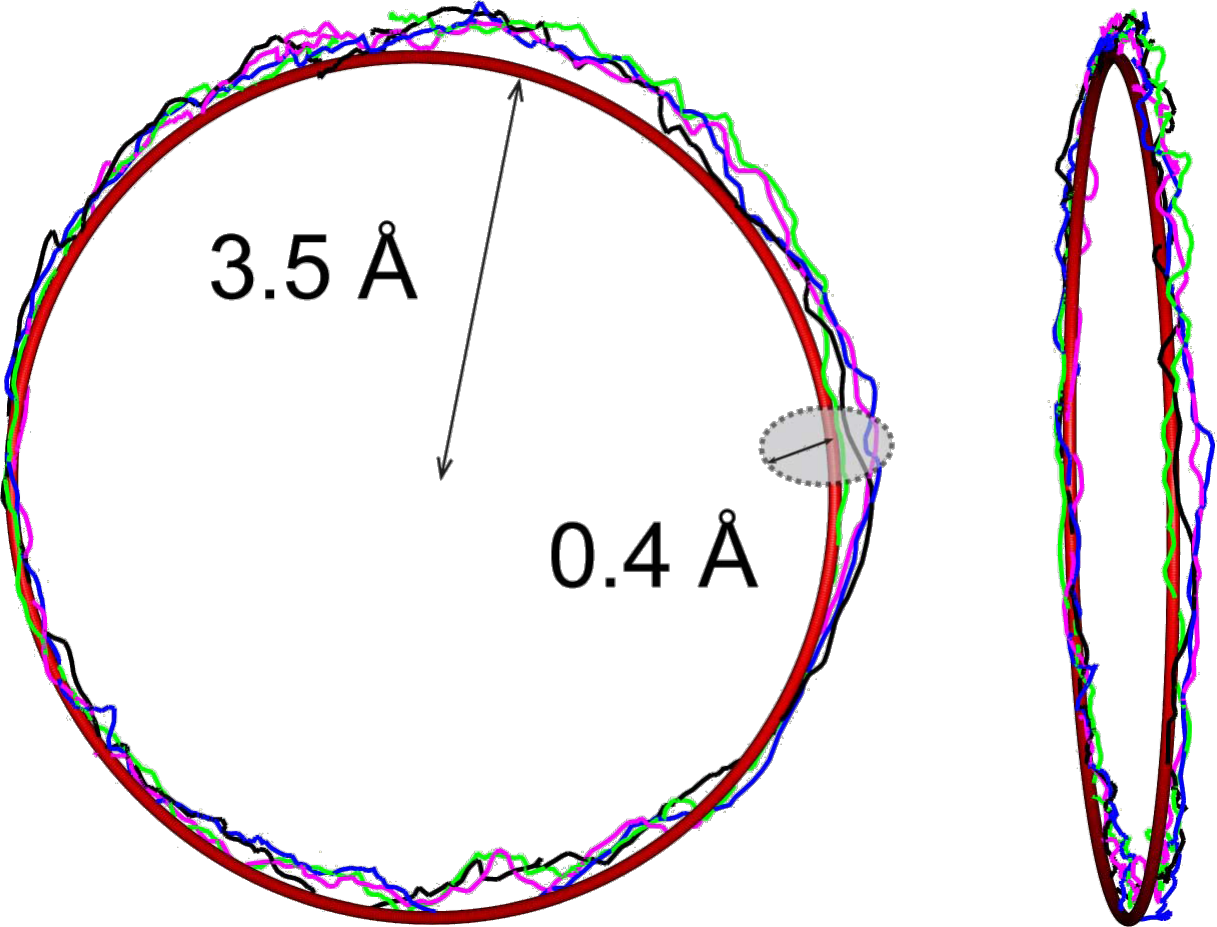
Precision of an arbitrary trajectory tracking in HCM with the visual feedback. The operator followed a circle with HCM for four times. Each attempt took one minute and the maximum deviation from the cycle was approx. 0.4 Å. Further details in text.

We begin the discussion of our results by characterising the precision of the implemented visual feedback system. The characterisation was performed in the following manner: First, a circle with a diameter of 7 Å oriented arbitrarily in 3D space was visualized in ORt. The operator, wearing ORt, tried to follow the drawn circle by moving the Apex device held in their hand. Four of such attempts are exhibited in Figure 3. Superimposing the original circle with a torus of minimal volume that encloses all hand-drawn trajectories we find that the maximum error of trajectory tracking was approx. 0.4 Å. The accuracy of the manual trajectory tracking depends on the movement speed of the operator’s hand and thus can be improved further. Note, however, that the oscillation amplitude of the NC-AFM tuning fork sensor (approx. 0.4 Å) directly adds to the error of the trajectory tracking and thus limits the achievable accuracy.

**Figure 4:**
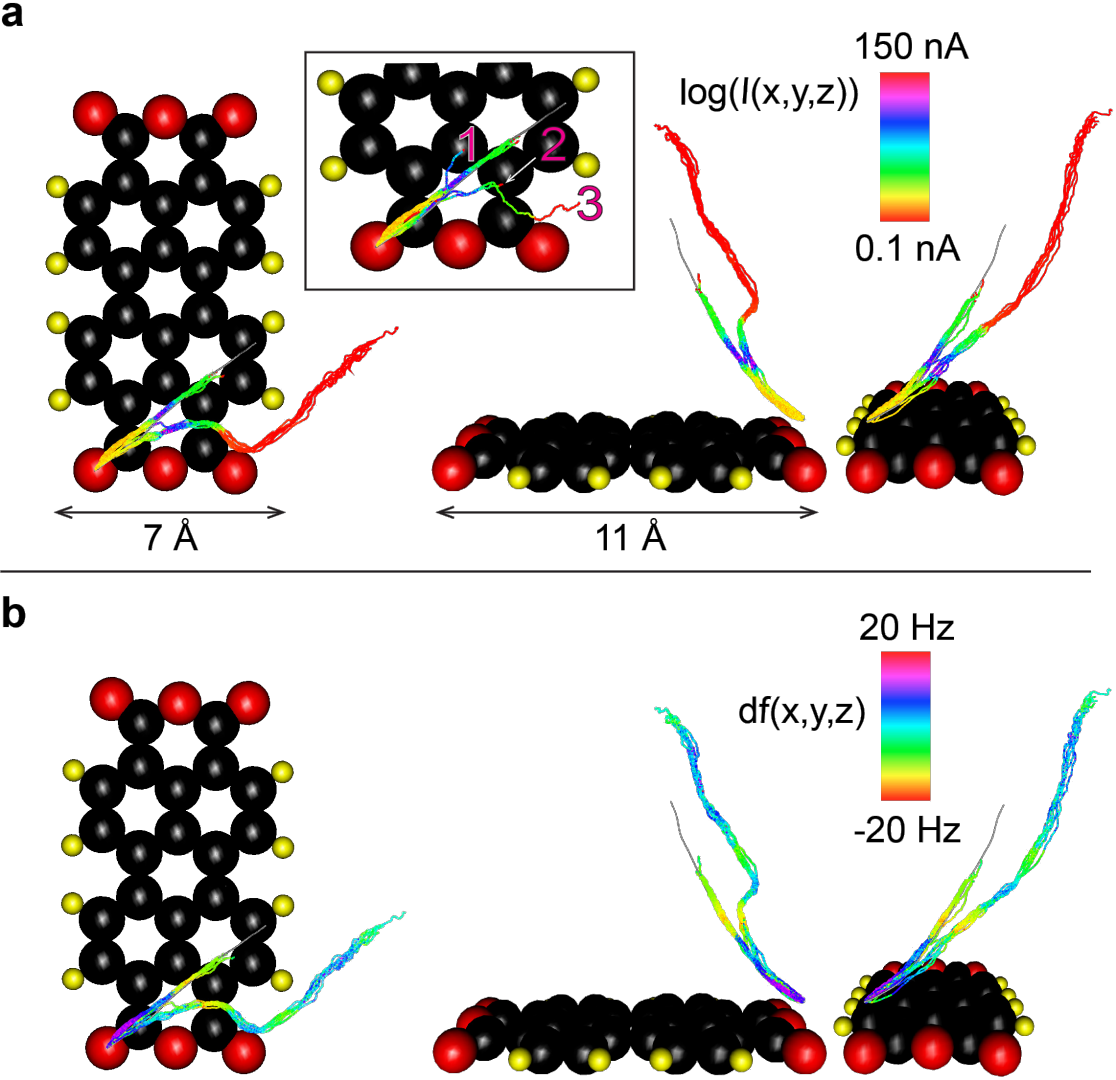
Manipulation trajectories recorded using HCM with the visual feedback. The inset in (a) shows three manipulation attempts (numbered by the sequence of their appearance) performed by the operator in the search of the kinked trajectory shown in (a) and (b). Top and side views of a) log(*I*(*x*,*y*,*z*)) and b) Δ*f*(*x*,*y*,*z*) trajectories recorded by pulling a single PTCDA molecule out of the PTCDA monolayer grown on Ag(111). Orientation of the molecule is shown in Figure 1. A section of the averaged trajectory obtained from [1] is shown in gray. Seven attempts following the averaged trajectory fail due to a premature loss of the tip–molecule contact. In contrast, eight attempts following a newly found kinked trajectory lift the molecule far from the surface without loosing the tip–molecule contact.

Further, we applied the extended HCM set-up to our model manipulation problem of [1], i.e., extraction of PTCDA from a monolayer adsorbed on Ag(111). First, by averaging all the successful tip trajectories from [1] we composed a single 3D trajectory. The averaged trajectory that is shown in Figure 4 was then plotted in ORt to serve as a guide for the operator. The plotted trajectory was rotated around the *z*-axis (perpendicular to the surface) to match the ORt view to the real azimuthal orientation of the chosen PTCDA molecule on the sample surface. As shown in Figure 4, in the correct orientation the projection of the averaged trajectory roughly follows the diagonal of the rectangular molecular backbone (for 3D images see Supporting Information File 1).

After the alignment of the ORt image, the manipulation process was started. First, using the standard options of our SPM software, we parked the tip directly above the oxygen atom of the PTCDA that was chosen for contacting (Figure 1). After the tip was stabilized, the STM current feedback loop was opened and the control over the tip position was passed to the operator. The operator contacted the molecule by moving the tip in a strictly vertical trajectory (*x*,*y* tip coordinates frozen) until a sharp jump of the *I* and Δ*f* bar indicators in the ORt display indicated establishing of the contact between tip and PTCDA (see Supporting Information File 2). After the contact was established the tip control was switched from the 1D (only *z*) to full 3D (*x*,*y*,*z*) HCM mode.

Using HCM the operator made seven attempts to follow the average trajectory. As Figure 4 shows, in all cases the contact to the molecule was lost after about *r* = 5 Å of tip retraction. The breaking of the contact was recognized by an abrupt simultaneous drop of the *I* and Δ*f* signals, which could be directly observed in the ORt display (see Supporting Information File 2). The early rupture of the tip–PTCDA contact indicated that the chosen tip trajectory was not optimal. The deficiency of the averaged trajectory can be explained by the fact that it was generated by averaging the datasets obtained by the extraction of PTCDA molecules that had different numbers of nearest neighbours [1]. Since we expect the intermolecular interactions in the monolayer to play a significant role, the tip trajectories that extract molecules from different intermolecular configurations most likely deviate from each other substantially. Thus, averaging them could produce a trajectory that is not optimal for any particular configuration, such as the one chosen in our present experiment (Figure 1). Another argument for a poor performance of the averaged trajectory may be supplied by assuming different tip structures. In other words, the tip used here could form a weaker bond to the molecule than the tip employed in [1]. However, the fact that the failure of the averaged trajectory occurred also after several tip-forming procedures, makes the second argument less convincing.

The failure of the averaged trajectory challenged us with the problem of identification of a new tip trajectory that is able to extract a PTCDA molecule from the chosen configuration. We show that HCM with ORt visual feedback allows the operator to identify the necessary tip trajectory very efficiently, making only a few manipulation attempts. The inset of Figure 4a exhibits three steps of the search process: In the first attempt (trajectory 1) the operator deviated laterally from the unsuccessful averaged trajectory. The first attempt failed and the contact to the molecules was lost rather early. The second attempt (trajectory 2) however took a different direction and there the contact to the tip was kept longer. In the third attempt (trajectory 3) the operator first followed trajectory 2 but then made another twist which finally enabled further lifting. Following trajectory 3, the operator sequentially lifted the molecule eight times, each time retracting the tip by up to 12 Å from the surface without a single failure of the tip–molecule bond.

It is important to note that in the course of the manipulation experiments in Figure 4 the molecule was never fully detached from the surface in order to prevent a tip structure change from happening, e.g., due to a jump of the extracted molecule onto the tip apex. After retracting the tip by 11–12 Å it was approached back to the surface and the tip–molecule bond was cleaved by carefully raising the junction bias to 0.1–0.5 V. In all cases after this voltage increase the molecule was re-deposited back to the substrate, precisely into its previous configuration within the monolayer. Using the described procedure we were able to contact and lift the same molecule with the same tip which, as Figure 4 shows (see also the interactive 3D models provided in Supporting Information File 1), resulted in a highly reproducible behaviour of *I*(*x*,*y*,*z*) and Δ*f*(*x*,*y*,*z*).

## Conclusion

In summary, we have extended our SPM HCM set-up by adding a visual feedback system based on Oculus Rift virtual reality goggles. Visual feedback helps the operator performing HCM to control their actions in real time by directly observing the executed tip trajectory in 3D. The system also allows the operator to follow any arbitrary preset 3D trajectories and execute controlled deviations from them. The typical error of the trajectory following was found to be about 0.4Å. The extended SPM HCM set-up was applied to the model problem of extracting of a single PTCDA molecule out of a PTCDA/Ag(111) monolayer. We demonstrated that the added visual feedback facilitates the systematic search for optimal manipulation trajectories. The kinked manipulation trajectory that was found in this work aided the uninterrupted lifting of the molecule from the surface. The stability of the established manipulation protocol resulted in the collection of a very reproducible *I*(*x*,*y*,*z*) and Δ*f*(*x*,*y*,*z*) data set that clearly exhibits interesting signatures of the molecular manipulation process and which opens up the prospect for systematic studies.

## Supporting Information

File 1 Screenshot Oculus Rift movie showing different stages of manipulation (watch subtitles for additional information).

File 2 Interactive 3D models of the data shown in Figure 4. In order to view it unpack and open either ’df.html’ (frequency shift) or ’I.html’ (logarithm of the current) file.

## References

[1] Green M F B, Esat T, Wagner C, Leinen P, Grötsch A, Tautz F S, Temirov R (2014). Beilstein J Nanotechnol.

[2] Rohlfing M, Temirov R, Tautz F S (2007). Phys Rev B.

[3] Kraft A, Temirov R, Henze S K M, Soubatch S, Rohlfing M, Tautz F S (2006). Phys Rev B.

[4] Giessibl F J (2003). Rev Mod Phys.

[5] Temirov R, Lassise A, Anders F B, Tautz F S (2008). Nanotechnology.

[6] Toher C, Temirov R, Greuling A, Pump F, Kaczmarski M, Cuniberti G, Rohlfing M, Tautz F S (2011). Phys Rev B.

[7] Fournier N, Wagner C, Weiss C, Temirov R, Tautz F S (2011). Phys Rev B.

[8] Wagner C, Fournier N, Tautz F S, Temirov R (2012). Phys Rev Lett.

[9] Kilian L, Hauschild A, Temirov R, Soubatch S, Schöll A, Bendounan A, Reinert F, Lee T-L, Tautz F S, Sokolowski M (2008). Phys Rev Lett.

[R10] 10Using ORt we found that it interfered with operation of the VICON system: In case a single point-marker is used with the VICON system and because ORt also uses multiple sources of infra-red light (fixed at a surface of the goggles) for positioning, the correct determination of the marker’s position becomes complicated. To overcome that problem we used VICON Apex device that is recognised by the VICON software as a 3D object with a unique shape which allows the VICON software to distinguish it clearly from the shapes created by the light sources installed on ORt.

