# Supplementary material for: Virtual reality visual feedback for hand-controlled scanning probe microscopy manipulation of single molecules
Source: Beilstein J Nanotechnol. 2015 Nov 16;6:2148–53. doi: 10.3762/bjnano.6.220 (PMC4660913; doi:10.3762/bjnano.6.220)
Supplement: File 2 — Interactive 3D models of the data shown in Figure 4. In order to view it unpack and open either ’df.html’ (frequency shift) or ’I.html’ (logarithm of the current) file. [file Beilstein_J_Nanotechnol-06-2148-s002.zip › 3Dmodel/df.html]

 
RGL model


Your browser does not support the HTML5 canvas element.


You must enable Javascript to view this page properly.

  
Drag mouse to rotate model. Use mouse wheel or middle button
to zoom it.

---

  
Object written from rgl 0.93.1098 by writeWebGL.
